# Evaluating vacant middle seats and masks as Coronavirus exposure reduction strategies in aircraft cabins using particle tracer experiments and computational fluid dynamics simulations

**DOI:** 10.1002/eng2.12582

**Published:** 2022-11-05

**Authors:** James S. Bennett, Seif Mahmoud, Watts Dietrich, Byron Jones, Mohammad Hosni

**Affiliations:** ^1^ Division of Field Studies and Engineering National Institute for Occupational Safety and Health, CDC Cincinnati Ohio USA; ^2^ University of Cincinnati Cincinnati Ohio USA; ^3^ Department of Mechanical and Nuclear Engineering Kansas State University Manhattan Kansas USA

**Keywords:** aircraft cabin, COVID‐19, mask wearing, MS2 virus, tracer particles, vacant middle seat

## Abstract

Aircraft cabins have high‐performance ventilation systems, yet typically hold many persons in close proximity for long durations. The current study estimated airborne virus exposure and infection reductions when middle seats are vacant compared to full occupancy and when passengers wear surgical masks in aircraft. Tracer particle data reported by U.S. Transportation Command (TRANSCOM) and CFD simulations reported by Boeing were used along with NIOSH data, to build nonlinear regression models with particle exposure and distance from particle source as variables. These models that estimate exposure at given distances from the viral source were applied to evaluate exposure reductions from vacant middle seats. Reductions averaged 54% for the seat row where an infectious passenger is located and 36% for a 24‐row cabin containing one infectious passenger, with middle seats vacant. Analysis of the TRANSCOM data showed that universal masking (surgical masks) reduced exposures by 62% and showed masking and physical distancing provide further reductions when practiced together. For a notional scenario involving 10 infectious passengers, compared with no intervention, masking, distancing, and both would prevent 6.2, 3.8, and 7.6 secondary infections, respectively, using the Wells–Riley equation. These results suggest distancing alone, masking alone, and these practiced together reduce SARS CoV‐2 exposure risk in increasing order of effectiveness, when an infectious passenger is present.

## Graphical abstract

Viral exposure reductions compared to full occupancy averaged 54% for the seat row where an infectious passenger is located and 36% for a 24‐row cabin containing one infectious passenger, with middle seats vacant. Analysis of the TRANSCOM data showed that universal masking (surgical masks) reduced exposures by 62% and showed masking and physical distancing provides further reductions when practiced together. For an entirely notional scenario involving 10 infectious passengers, compared with no intervention, masking, distancing, and both would prevent 6.2, 3.8, and 7.6 secondary infections, respectively, using the Wells–Riley equation.

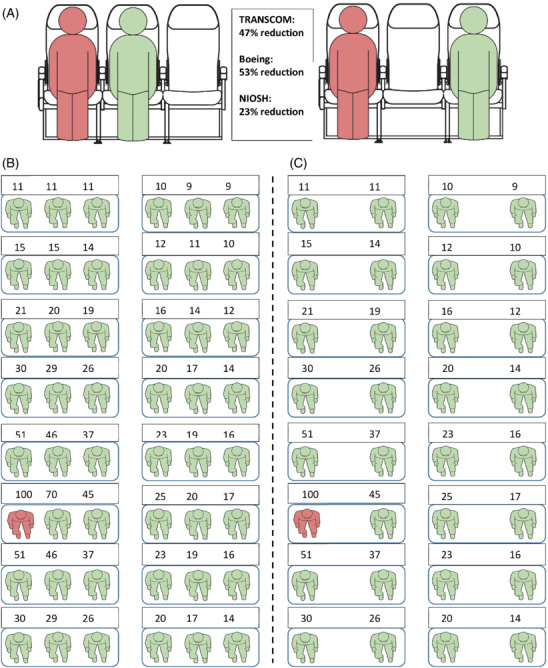

## 1 INTRODUCTION

Aircraft cabins are served by high‐performance ventilation systems. They also typically hold many persons in close proximity for long durations. Research suggests that seating proximity on aircraft is somewhat associated with increased risk for infection with SARS‐CoV‐2, the virus that causes COVID‐19, but the importance of seating in the context of other factors is not clear. As reported by Murphy et al.,^1^ 13 infections among those onboard were later confirmed by nasopharyngeal swab PCR. This 7.5‐h flight had only 17% occupancy. No index passenger was identified, and nine of the 13 infected persons reported wearing masks. A source proximity effect was identified by Khanh et al.^2^ in finding that most of the 15 confirmed cases and the known index passenger were seated in the business class section during a 10‐h flight. An association of COVID‐19 infection, seating proximity, and mask behavior was reported by Toyokawa et al.^3^ for a 2‐h flight within Japan. The authors calculated adjusted odds ratios of 7.29 for no mask, 3.0 for partial wearing of a mask, and 7.46 for being seated within two rows of the index passenger.

A well‐known study by Olsen et al.^4^ during the Severe Airborne Respiratory Syndrome (SARS) epidemic reported seating locations of probable secondary infections and the index passenger, in one of three flights that were tracked due to one or more passengers having tested positive for SARS. In 2013, after the H1N1 epidemic and during the Middle East Respiratory Syndrome (MERS) outbreaks and ongoing efforts to prepare for a possible influenza pandemic, a National Institute for Occupational Safety and Health (NIOSH) study fit closely the number of infections reported in Olsen to a linear regression, with distance as cabin rows from the index passenger the independent variable. This finding along with regression models of tracer studies conducted in cabin mock‐ups at the University of Illinois and Kansas State University demonstrated that air contaminants, including droplets, travel several seat rows beyond the source row.^5^ Following on in 2018, a conference paper^6^ showed that bacteriophage (MS2) droplets emitted from a spray bottle into Boeing 767 and 737 mock‐ups dispersed like other particle tracers. The fluid aliquots collected in a bioaerosol liquid impinger were evaluated for viral particle presence by plaque assay. The number of plaque‐forming units (PFUs), considered proportional to the airborne concentration of viable virus, were fit to an exponential decrease curve using radial distance between source and measurement location.

Aircraft cabin environmental control systems are designed and operated to deliver amounts of clean air per occupant that conform to various standards, including requirements set by the Federal Aviation Administration and Recommendations of the American Society of Heating, Refrigeration, and Air‐Conditioning Engineers.^7^, ^8^ Under these conditions, most virus particles are removed within several seat rows from a source on an aircraft, and the recirculated portion of the air supplied to each passenger has passed through high efficiency particle air (HEPA) filters.^9^ Physical distancing is difficult on crowded flights; and, sitting within 2 m (6 ft) of others, sometimes for hours, might increase risk for SARS‐CoV‐2 exposure. To reduce this risk, CDC issued an order in January 2021 that required the wearing of masks in travel settings (on conveyances and at US transportation hubs) to prevent spread of COVID‐19, including all passengers and crew on aircraft traveling into, within, or out of the United States,^10^, ^11^ and recommends all people be up to date with COVID‐19 vaccines before travel.^12^ On April 18, 2022, CDC stopped enforcing the mask order but continued to recommend masking in public transportation settings.^12^ Prevalence of COVID‐19 among the flying public can be estimated from international travelers arriving by air in Canada. From February 21, 2021 to July 4, 2021, 0.90% of all arriving passengers tested positive; from July 5 to September 16, 0.90% of partially or unvaccinated passengers tested positive; and, from September 10 to November 6, 0.64% tested positive. For fully vaccinated passengers, the rate dropped to 0.21% for July 5 to September 16 and to 0.15% from September 10 to November 6 but then increased to 5.0% for November 28, 2021 to January 22, 2022.^13^

As the SARS‐CoV‐2 pandemic was taking hold in early 2020, many air carriers created socially‐distance seating environments, notably Delta Airlines, which blocked the selection of middle seats and capped seating in every cabin class.^14^ This carrier maintained the practice through April 30, 2021, longer than any other U.S.‐based airline.^15^ Earlier, NIOSH had re‐analyzed the MS2 bacteriophage tracer experiments to estimate the effect of leaving middle seats vacant compared to full occupancy and issued a short report in the Morbidity and Mortality Weekly Report (MMWR), finding that relative exposure to the bacteriophage droplets in vacant middle seat (VMS) scenarios was reduced by 23% to 57%.^16^ A knowledge gap—not accounting for mask wearing—was identified in press coverage of the report.^17^ While the MMWR report did not evaluate the effect of mask wearing (masking) or the interaction of VMS and masking, it reported VMS only as a relative exposure reduction that would act independently from masking if lack of interaction, for example, changing the droplet size distribution, could be reasonably assumed. This assumption is explored in the current study. A New York Times piece stated that the infection risk was low due to ventilation and filtration,^17^ and we reference the popular press article here because it captures a view held widely by researchers and the air travel industry. A contrasting view is that higher density^18^ and longer duration^19^ of occupancy compared to many indoor environments complicate this characterization.

The MMWR report was not alone in predicting lower exposure without adjacent seating. Horstman and Rahai^20^ assayed this scenario using CFD in a single‐aisle, three‐on‐each side seating arrangement and report a 40% infection risk decrease due to increased supply air per occupant and specific flow patterns. Pavlik et al.^21^ compare various seating distance and masking implementations while illustrating the dependence of pattern optimality on occupancy level and spatial risk model. VMS may not be a particularly efficient rule. Barnett and Fleming^22^ calculated, from extensive case searches and probabilistic modeling, COVID‐19 infection risk estimates of 1/1000 and 1/6000 for a 2‐h flight, when full during highest and when half‐full during lowest pandemic infection periods, respectively.

In October of 2020, the TRANSCOM Report that detailed aerosol tracer experiments onboard functioning (including flying) Boeing 767 and 777 aircraft was released publicly,^23^ and a corresponding peer‐reviewed journal article was published in December of 2021.^24^ This large work included hundreds of trials with and without the source manikin wearing a three‐ply surgical mask. The data were used by Wang et al.^25^ as a seat‐specific exposure map to avoid the much criticized completely mixed assumption in their application of the Wells–Riley equation. Authors from Boeing released a pre‐print and published a journal article documenting CFD simulations of a Boeing 737 cabin that tracked a realistic droplet distribution from several source seat locations under varying ventilation and thermal conditions.^26^

In all aspects other than release and measurement of a live virus, the TRANSCOM paper by Kinahan^24^ provides a much greater wealth of cabin data than the Lynch paper.^6^ The Zee paper^26^ is unique in reporting CFD results at all seat locations in the computational domain. The strength of these data sources and the manner the VMS MMWR report was received motivated substantially extending the VMS work to include the Kinahan and Zee datasets. The current article describes this effort, including characterization of masking, and examines relative exposure reduction in the context of a range of probable infections, using a simplified form of the Wells–Riley equation.

## 2 METHODS

### 2.1 Methodology

Understanding the effects of seating distance and masking requires adequate approximation of the spatial and temporal behavior of respired droplet clouds that might contain infectious virions, in varying cabin environments. Methodological choices are abundant, and the complexity of the problem is beyond all practical avenues. From the always wrong and ever useful completely mixed model to the direct numerical simulation of all turbulence scales in CFD, all choices lack some accuracy in either the physics or the boundary conditions. Within this span are models that fuse fundamental scientific insights with situational specifics to illuminate the most important features. The analysis of tracer studies belongs to this functional middle, with experiments being useful to the extent the tracer and setup represent the material and environment of interest. Transport and diffusion models are classical residents of the middle ground having been developed according to Hanna,^27^ by Taylor in 1921 and Richardson in 1923.

Transport and diffusion (T & D) approaches were applied in Hanna^28^ to the TRANSCOM data to investigate the variable near‐field behavior of the manikin respiratory cloud. The analysis emphasizes the size of the dynamic cloud toward estimating concentration within, for a given particle release mass or number. Lin et al.^29^ report measured velocities in the seating area of a Boeing 777 as averaging approximately 50 ft/min (0.25 m/s) and a turbulence kinetic energy of 0.01 m^2^/s^2^ at the center node in a Boeing 767 CFD simulation. Han et al.^30^ analyzed the longitudinal (aisle‐direction) vortical structures, whereas cabin flows are often assumed to be two‐dimensional, with latitudinal vortices driven by the overhead horizontal supply jets.

Venkatram and Weil^31^ describe application of the diffusion, “K‐theory,” piece of T & D to indoor spaces for virus dose estimation. The tracer analyzes in the current study use no physics other than choosing an exponential or power law decrease with distance to model the data. Still, this method shares with K‐theory its most simple model—the isotropic decrease with radial distance from a point source—summarized in Venkatram and Weill as a single equation with empirical parameters, without mentioning aircraft cabins as a possible application.

(1) $$\text{Dose}=\frac{1}{Kr}.$$

Two considerations may justify an isotropic fit to tracer data in the current study. Exposure estimates are used only in the relative sense, that is, comparing seat B to seat C, rather than in the absolute sense where exposure at seats B and C are reported as certain values. More fundamentally, different methods seem to yield different results in the passenger aircraft cabin environment, where the boundaries of primary and secondary vortical structures are somewhat unstable, so that a highly averaged characterization is a practical path to insight.

All exposure analyzes in the current study used the dimensionless relative exposure, *E*_*R*_, defined here as the ratio of two exposure measurements with units. For example, the exposures in the Boeing CFD results were reported by those authors as percent of released mass; so, the unit is “% of released mass,” expressed this way for clarity, as percent is itself a sort of dimensionless quantity. In comparing exposures at seat C to seat B, we use the ratio *E*_*C*_ */E*_*B*_, which is unitless. We write a %‐reduction at seat C relative to seat B as 100%(1−*E*_*C*_ */E*_*B*_). Whether using plaque forming units, micrograms, or % of particle number released, the units disappear in the ratio. The values in the original units reported by the study authors were used here in the regression analyzes; however, the resulting predicted exposures at specific distances were only used in comparisons, that is, dimensionless ratios or %‐reductions.

### 2.2 Study structure

The study had four phases. In the first phase, data from three sources were analyzed by regression analysis: a bacteriophage MS2 virus dispersion study that measured broth droplets by plaque assay in aircraft cabin mock‐ups^6^, ^16^; the TRANSCOM study tracer data made publicly available during the pandemic^23^, ^24^; and Boeing CFD simulations conducted in response to the pandemic.^26^ In the second phase, the nonlinear regression models built from these datasets were used to estimate the reduction in aerosol concentration as distance increased, from the adjacent seat distance of 0.5 m (1.6 ft) to the adjacent + 1 distance of 1 m (3.3 ft). Confidence intervals were calculated at these locations from a linear regression on the log transformed variables followed by exponentiation. The models were applied to conceptual aircraft cabin seating scenarios to simulate the reduction in exposure resulting from VMS.

In the third phase, the TRANSCOM data were divided into unmasked and masked source groups for statistical comparison. In the fourth phase, these intervention analyzes were put in the context of hypothetical infectivities, which yielded estimates of the number of infected passengers, and application of a random factor demonstrated a variety of possible infection patterns.

#### 2.2.1 Phase 1: Aerosol decrease by nonlinear regression on distance

The current analysis used all TRANSCOM data that were collected in the coach cabin of the functioning B767 aircraft, with the source manikin unmasked, a total of 758 datapoints. In the power law regression, four interpolated data points closer to the source than the closest actual measurement location were added to characterize more completely the exposures very near the source. The actual particle count measured at 0.50 m (1.6 ft) from the manikin's mouth was used as the boundary condition to estimate closer values, using the notion of concentration, *C*(*r*), decrease as the inverse of radial distance, *r*, from a point source, or spherical diffusion.^32^ The inverse distance relation also appears in classical jet theory. According to Schlichting,^33^ the centerline velocity of a round, free jet (laminar or turbulent) entering a quiescent fluid decreases as *1/x*, where *x* is the distance along the jet centerline away from the origin, and the jet width increases as *x*. Since the circular jet width increases as *x*, the cross sectional area, *A*, increases as *x* ^*2*^, and the volumetric flow rate, *Q*, increases as the product of the centerline velocity and the area, or (*1/x*)(*x* ^*2*^) *= x*. The concentration is then proportional to the mass flow rate from the source divided by the volumetric flow rate of the jet, and the ratio of concentrations at different distances is:

(2) $$\frac{C_{2}}{C_{1}}=\frac{\frac{\dot{m}}{Q_{2}}}{\frac{\dot{m}}{Q_{1}}}=\frac{Q_{1}}{Q_{2}}=\frac{x_{1}}{x_{2}}.$$

The inverse distance behavior is predicted also by uniform eddy diffusivity models widely used in workplace exposure modeling.^34^ The relative exposure, *E*_*R*_, would in this case be thought of as the ratio of *E*_*D2*_ */E*_*D1*_ and this would be equal to *C*(*r*_*2*_)*/C*(*r*_*1*_) regardless of the concentration unit. The issue here is that the respiratory plume is certainly not isotropic, and it does not enter a quiescent fluid in the ventilated aircraft cabin, although the cabin velocity is much lower than the core velocity of a cough. Offered for consideration is that the harm may be minimal, because to the extent the emission jet is entrained at some distance by cabin flow and spreads due to shear, it loses its directional organization. In this sense, all roads lead to or near the Damascus of 1/*r*.

The nonlinear (power law) regression analysis of the Boeing CFD data used as the dependent variable, particle mass at individual seats as a percentage of the total released by a simulated coughing passenger, with radial source distance as the independent variable. The CFD cabin geometry included five rows of a 737 aircraft for a total of 29 seats other than the source seat. Nine simulations that varied source location, ventilation rate, and relative humidity were reported for a total of 261 datapoints.

#### 2.2.2 Phase 2: Exposure reduction with vacant middle seats

The present study and the previous NIOSH publication^16^ evaluated the exposure reduction due to vacant middle seats versus full cabin occupancy, by two methods. The first considered only the extra distance between passengers. Here, regression models of all datasets estimated exposure as a function of distance throughout the cabins. They were applied to the adjacent distance and to one seat further away from an infectious passenger to gage the relative reduction. The NIOSH data had only 17 measurements, which were fitted to an exponential decrease function with distance.

We calculated the average relative exposure, ${\bar{E}}_{R}$, across the three datasets by first dividing each regression function by what could be called its amplitude constant. As examples showing both exponential and power law functions,

(3) $$E=G\exp\left(-HD\right)\to \exp\left(-HD\right)\text{or}E=GD^{-H}\to D^{\text{–}H},$$

where *G* and *H* are nonnegative constants and *D* is distance in the original units. The average relative exposure is then something like:

(4) $${\bar{E}}_{R}=\exp\left(-H1D\right)+\exp\left(-H2D\right)+D^{‐H3}.$$

The VMS distance effect was also evaluated more directly by comparing source‐adjacent to two‐seats‐distant (adjacent + 1) exposures. The NIOSH MS2 data contained no adjacent + 1 samples. The TRANSCOM data followed a lognormal distribution and had six (adjacent, adjacent + 1) pairs of measurements. These occurred in the B777 cabin with a four‐seat section in the middle, seats DEFG. The manikin was in seat E. The adjacent seat was F, and adjacent + 1 was G. These 12 values were log transformed, and the mean difference in the pairs was exponentiated to find the reduction ratio. The Boeing CFD data contained nine source adjacent solution locations and eight adjacent + 1 locations. These 17 normally distributed values were analyzed by one‐sided comparison of means with pooled variance. Analyzes used Tukey's studentized *t*‐test.

The second approach combined the distance effect predicted by the regression models with the reduced total exposure due to fewer passengers that the distance effect is applied to, as these are inseparable in realistic arrangements of infectious passengers and other passengers. This confounded reduction effect was estimated by summing the exposures of the passengers who are present, as in Equation (5):

(5) $$\frac{R}{100\%}=1-\frac{\sum_{\mathrm{VMS}}E_{i}}{\sum_{\text{FULL}}E_{j}}$$

where *E*_*i*_ are the individual exposures of the passengers present with VMS and *E*_*j*_ are the exposures with full occupancy. Because the individual exposures depend on distance from the source passenger, Equation (5) represents both the distance effect and reduced occupancy.

However, as passengers not on one flight might be on another, a system of flights was included in the vacant middle seat analysis to examine whether exposure is simply being transferred rather than reduced. To do this we consider the average exposure, *E*_*P*_, per passenger for all like aircraft, where *E*_*PF*_ and *E*_*PVM*_ refer to full and vacant middle seat scenarios. Equation (6) estimates *E*_*P*_ using the probability *P* that any occupant is infectious, the number of passengers, *N*, and the total exposure *I*_*P*_ generated by one infectious passenger:

(6) $$E_{P}=\frac{\mathrm{PN}I_{P}}{N-\mathrm{PN}}=\frac{\text{Total infectious exposure}}{\text{Total passengers other than sources}},$$

This equation simplifies to:

(7) $$E_{P}=\frac{PI_{P}}{1-P},$$

which indicates that the average infectious exposure, for example the average exposure under a VMS policy, does not depend on how many passengers or flights are considered. The reduction in average exposure is then shown by Equation (7) to be simply the relative reduction in total exposure. Since *I*_*P*_ includes the effect of distance between source and receiver, the calculation combines the effects of reduced occupancy and increased distance.

(8) $$\frac{R}{100\%}=\frac{E_{\mathrm{PF}}-E_{\mathrm{PVM}}}{E_{\mathrm{PF}}}=\frac{\frac{PI_{F}}{1-P}-\frac{PI_{\mathrm{VM}}}{1-P}}{\frac{PI_{F}}{1-P}}=\frac{I_{F}-I_{\mathrm{VM}}}{I_{F}}.$$

This approach assumes a vacant middle seat policy does not affect the number of passengers in the system, or the fraction who are infectious, and that any infectious individuals are randomly distributed among the flying public.

#### 2.2.3 Phase 3: Effect of universal masking

The exposure reduction provided by masking was analyzed by itself and in combination with physical distancing. The extensive TRANSCOM data on source masking was used to assess the size of the effect and interactions of masking and distancing. All trials, where the masked source and the unmasked source were used under the same experimental conditions, were included in the analysis. Each source type had an *N* of 2757, for a total of 5514 observations. The particle counts were log‐transformed due to large positive skew in the distribution and grouped by unmasked and masked source. The difference in log means and 95% two‐sided confidence interval were formed using Tukey's studentized *t*‐test with pooled variance, before exponentiating. Masking data in the literature were also examined to form a range of possible protections.

#### 2.2.4 Phase 4: Infectivity and probability

The present study applied notional exposure thresholds for viral infection. The authors believe this framework is useful for understanding potential effects of protective measures, even with infection rate uncertainty, especially since infectivity seems to depend on the specific virus strain. Exposure thresholds do not capture the variability of infection among a group of people with the same exposure. Therefore, a form of the Wells–Riley equation was used to predict the probability of infection:

(9) $$P=1-\exp\left(-aE_{R}t\right),$$

where *E*_*R*_ is the relative exposure, *t* is the duration of the exposure, and a is an infection coefficient that includes both source strength and virus infectivity. Here, as elsewhere in these analyzes, the focus is %‐reductions provided by VMS and universal masking, and relative exposure is sufficient for these determinations, because the %‐change in relative exposure is identical to the %‐change in absolute exposure. However, source strength, infectivity, and exposure duration do affect the infection probability as shown in Equation (9). Therefore, several notional values of the product of factors, at, were used in the comparisons. These values were adjusted to predict 1.0, 10.0, and 25.0 infections per infectious passenger when one infectious passenger is present, under full occupancy and no masking (Table 2). With two infectious passengers, predicted infections, per infectious passenger were reduced slightly to 1.0, 9.9, and 23.2 due to exposure from each source infecting the same passenger. The calculations were done for a 24‐row single aisle cabin.

To account in some way for the natural variabilities not captured by the methods described above, the infection probabilities were compared to a random number ranging from 0 to 1, using the RAND function in Excel. An infection was counted if and only if the infection probability was above the random number. Specific infection maps were thereby generated that converge to the Wells–Riley predictions only in an average sense. All analyzes included 24‐rows of a single‐aisle cabin with six seats per row.

## 3 RESULTS

### 3.1 Aerosol decrease by nonlinear regression on distance

**FIGURE 1:**
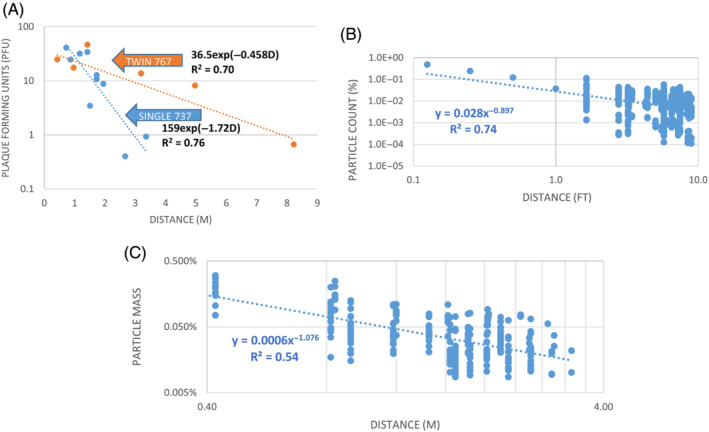
(A) Association between the number of plaque‐forming units and the distance between MS2 droplet source and sample locations, by single‐aisle (blue) and twin‐aisle (orange) cabin scenarios. The best fit exponential decay for both cabins combined was 27.5exp(−0.511D). (B) Decrease in particle counts with distance from exhaling manikin, as a percentage of number released in the TRANSCOM Study. The four values closest to zero distance are estimated from the smallest distance (1.6 ft) datapoints, as increasing by 1/*D* where *D* is distance. (C) Particle mass at individual seats as a percentage of the total released in the five‐row Boeing CFD simulations, for 29 seats and 9 trials that varied source location, ventilation rate and relative humidity (*N* = 261)

In the NIOSH data, the number of plaque‐forming units (PFUs), evidence of the presence of viable virus, declined exponentially (R^2^ = 0.70) with increasing distance between source and sample locations (Figure 1A). These virion data were fitted to an exponential regression equation of the form, *E = Ge* ^−*HD*^, with number of PFUs (*E*) as the dependent variable and the distance between spray and sampling locations (*D*) as the independent variable. *E* in general denotes the exposure in the units as reported in the source of the data—plaque forming units (NIOSH), % of particle count released (TRANSCOM), or % of particle mass released (Boeing CFD).

The TRANSCOM tracer data for an unmasked source, when estimates based on jet theory of the particle count closer to the manikin's mouth than the nearest measurement point were included, fit a power law model, *E = G D* ^−*H*^ (R^2^ = 0.74), because this function transitions faster between the rapid drop very close to the source and the more gradual decline, farther away (Figure 1B). Fluorescent microsphere count (*E*) was the dependent variable, and the distance between release from the manikin's mouth and the locations of the fluorescent particle counters (*D*) was the independent variable.

The Boeing CFD results—particle mass at individual seats (Figure 1C)—fit less closely (R^2^ = 0.54) a power law model of the form *E = G D* ^−*H*^ with *E*, the particle mass at the face of simulated passengers when the emitted, evaporating droplets were tracked using a Lagrangian discrete phase model and *D* the distance between droplet source (sinusoidal to simulate breathing) and the passenger faces. In all cases, *G* and *H* are the parameters determined in the statistical calculations performed using the trendline feature in Microsoft Excel, as was the coefficient of determination, R^2^.

It is important to understand that these equations reflect the average reduction with distance and do not precisely predict the exposure at a specific seat location. Interestingly, the power law exponent, negative 1.08, resulting from the regression analysis of the Boeing CFD data is quite close to the spherical diffusion and jet theory exponent in Equation (2): *D* ^*−1*^. Extensive tracer gas experiments, like those used to form the regression equations, show that local exposure at a specific site is often anisotropic and governed by local airflow patterns.^35^ The location of an infectious person in an aircraft will not be known. Thus, assessments must be based on average effects and not specific local effects.

**FIGURE 2:**
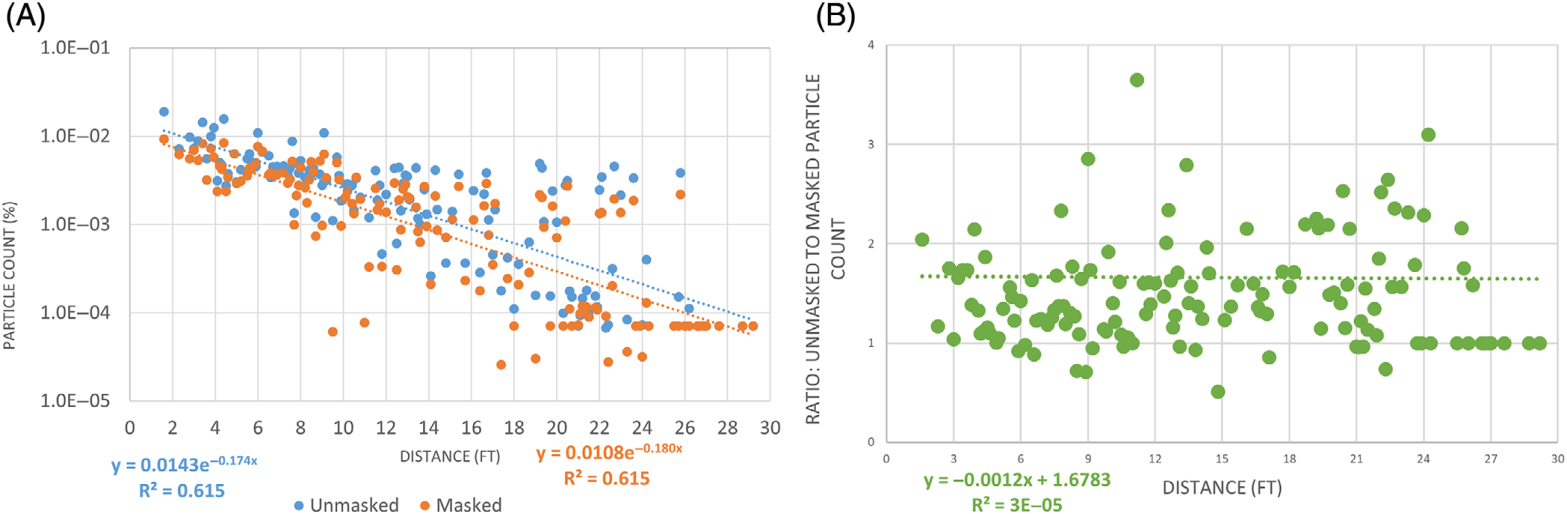
(A) Particle counts as a percentage of total released for unmasked (blue) and masked (orange) source manikin, as means of all measurements by a radial distance from the source manikin in the TRANSCOM study. (B) Ratio of unmasked to masked exposure remains essentially constant with distance

The TRANSCOM dataset, when separated into masked and unmasked source data and fitted to exponential regression equations of the form, *E = G e* ^−*HD*^, showed very similar fits and functions with and without masking, and the ratio of masked and unmasked data showed no trend (Figure 2).

### 3.2 Physical distance

**FIGURE 3:**
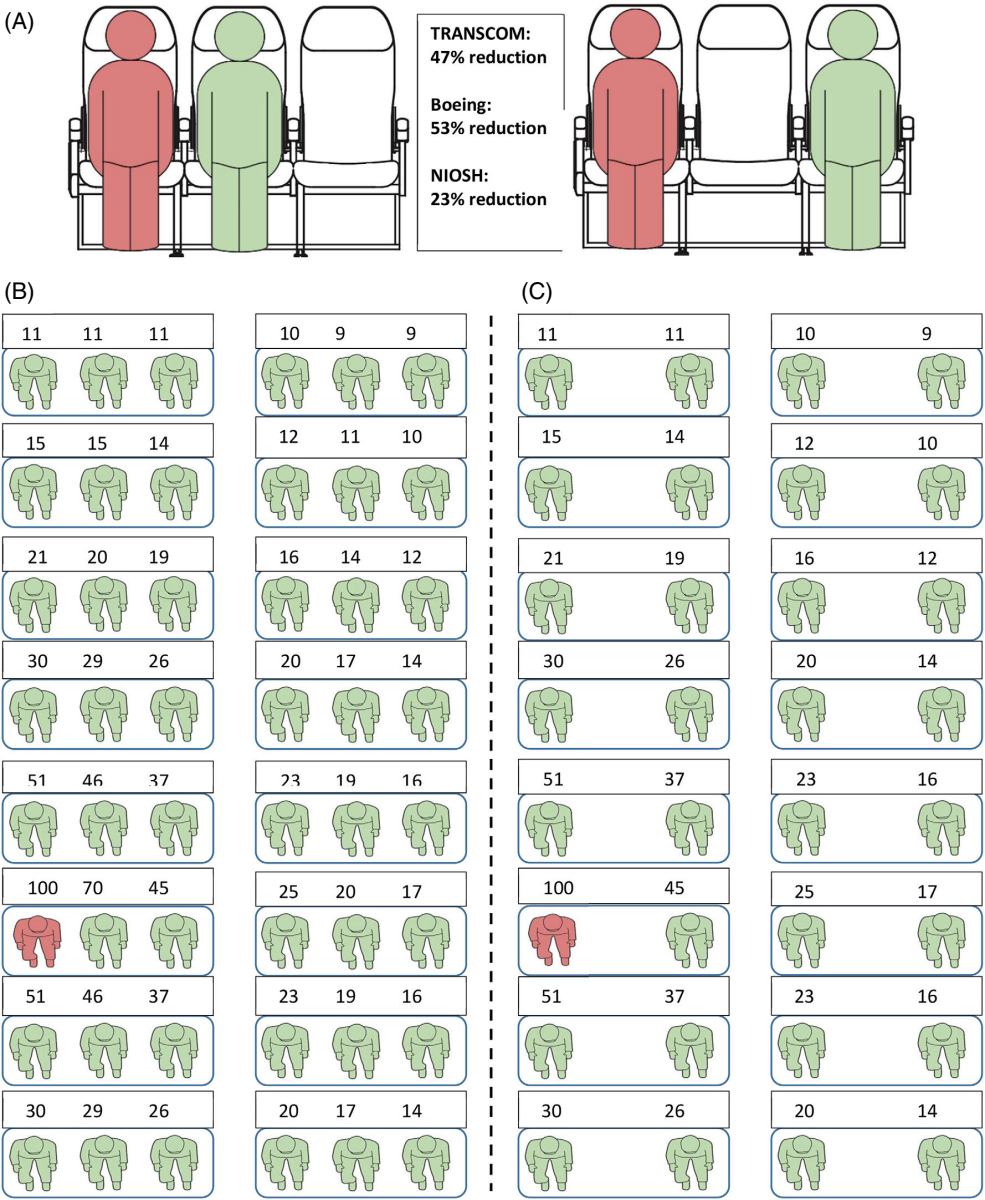
(A) Exposure reduction through increased distance from a potentially infectious passenger (red), in a window or aisle seat, for one adjacent passenger (green) moving one seat farther away. The same reduction occurs if the middle passenger is the infectious one and moves one seat away. (B) Relative exposure map (NIOSH, Boeing, and TRANSCOM averaged) for one infectious passenger in a window seat. (C) The same map with middle seat passengers and their exposures removed. Eight rows are shown and the analyzes were performed on 24 rows.

The first approach predicted a 23% exposure reduction (NIOSH MS2 data) by moving an adjacent passenger one seat further away from an infectious passenger. Using TRANSCOM tracer particle data, the single passenger reduction was 47%, and for the Boeing CFD simulations, it was 53% (Figure 3A). However, in direct individual seat comparisons, the average reduction was 41% for TRANSCOM and 20% for Boeing CFD data. Table 1 summarizes the statistical results including confidence bands, evaluated at the adjacent seat source distance and the adjacent + 1 seat distance, for the regression predictions. The exposure reductions for the adjacent + 1 distance compared to the adjacent distance were calculated using these predicted values. The adjacent and adjacent + 1 seat exposure bands overlapped in the NIOSH MS2 data, and this is attributed to the small sample size. A countertrend *increase* in exposure would then be expected in some cases. The models that used aircraft industry study data showed larger reductions in exposure when middle seats are vacant than did the models based on NIOSH data, perhaps because the NIOSH measurement locations, spread sparsely throughout the cabin, resulted in more spatial averaging in the regression analysis. The range of reductions, formed using different methods in three independent studies, is small enough to provide some confidence that the distance effect is well understood.

**TABLE 1:** Seat exposure reduction statistics by regression and means comparison

Data source	NIOSH MS2	TRANSCOM	Boeing CFD
Unit of measure	Plaque forming units	Particle count as % of release	Droplet mass as % of release
Equation	Single aisle 159exp(−1.72D) R^2^ = 0.76 Twin aisle 36.5exp(−0.458D) R^2^ = 0.70 Combined *y* = 27.528 e^−0.511*x*^ R^2^ = 0.4697	*y* = 0.028*x* ^−0.897^ R^2^ = 0.74	*y* = 0.0006*x* ^−1.076^ R^2^ = 0.54
Exposure at 0.5 m (1.6 ft) and 90%‐CI	21.3 [10.6, 43.0]	0.0184 [0.0131, 0.0258]	0.119 [0.101, 0.140]
Exposure at 1 m (3.3 ft) and 90%‐CI	16.5 [8.78, 31.0]	0.00985 [0.00821, 0.0118]	0.0565 [0.0520, 0.0615]
% Difference calculated from regression predictions at 0.5 m (1.6 ft) and 1 m (3.3 ft)	−22.6	−46.3	−52.6
% Difference as mean exposure reduction for adjacent + 1 compared to adjacent One‐sided *t*‐test	No adjacent measurements	−41.0 Hypothesis: adjacent + 1 seat exposure is lower than adjacent seat exposure. *p* = 0.0280 *N* = 12	−19.8 Hypothesis: adjacent + 1 seat exposure is lower than adjacent seat exposure. *p* = 0.0946 *N* = 17

### 3.3 Physical distance and occupancy

Using Equation (5), the VMS scenario compared to full occupancy reduced average exposure by 47%, 56%, and 59% for the row of an infectious passenger and by 36%, 36%, and 35% for a 24‐row cabin, using NIOSH, TRANSCOM, and Boeing data, respectively (Figure 3B,C). The VMS effect is not much higher than the 1/3 reduction in passengers in the cabin, when considering the cabin average; however, the effect is larger when closer to an infectious passenger.

### 3.4 Masking

The TRANSCOM study dataset of masked source and unmasked source (each *N* = 2757) particle counts at individual seats (sampling locations) provided an average reduction for masking of 38.2% with 2‐sided, 95‐% confidence interval [32.2%, 43.8%]. There were a small number of observations, where the particle count was higher for the masked than the unmasked source. Note that only the source manikin was masked. An estimate of the equivalent reduction under universal masking, *R*_*UM*_ is to treat source masking and receiver masking as equal and independent reductions, *R*_*S*_ and *R*_*R*_. According to Equation (10), the universal masking reduction is approximately 62%. Applying this equation to the confidence limits gives [54.0, 68.4] %.

(10) $$R_{\mathrm{UM}}=100\%\left\{1-\left(1-\frac{R_{S}}{100}\right)\left(1-\frac{R_{R}}{100}\right)\right\}=100\%\left\{1-\left(1-\frac{38.2}{100}\right)\left(1-\frac{38.2}{100}\right)\right\}=61.8\%.$$

Figure 2 shows that the trendlines for the unmasked and masked particle counts are essentially parallel and indicates that masking is well modeled as a constant reduction factor. Therefore, masking effectiveness results from the literature are reasonably applied to physical distancing reductions as independent relative reductions in exposure according to, once again, the independent reductions formula:

(11) $$R_{T}=100\%\left\{1-\left(1-\frac{R_{D}}{100}\right)\left(1-\frac{R_{\mathrm{UM}}}{100}\right)\right\},$$

where *R*_*T*_ *, R*_*D*_, and *R*_*UM*_ are the %‐reductions in total, from distancing and from universal masking. Hao et al.^36^ found particle reductions ranging from 10% to 95% when testing various masking materials themselves when not worn. The single‐row and 24‐row average physical distancing reductions, combined with the surgical mask effectiveness of 62% calculated from the TRANSCOM data, provided 82% and 75% reductions, respectively.

### 3.5 Exposure thresholds

**FIGURE 4:**
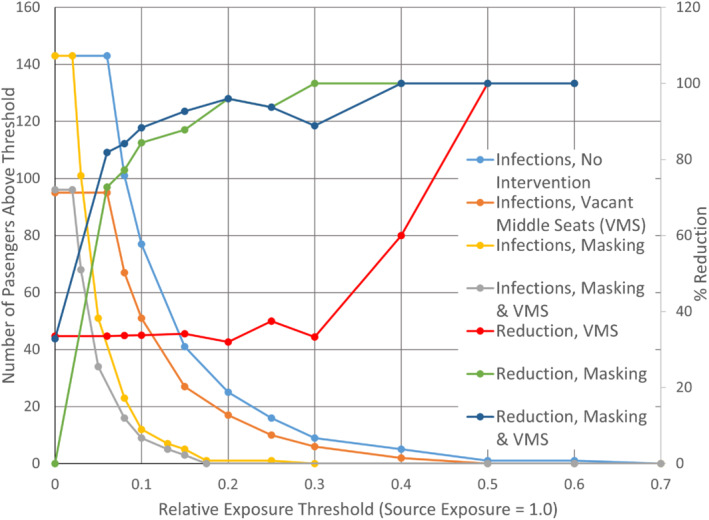
Number of passengers above a notional infection threshold for infection (left axis) and the %‐reduction in these numbers (right axis), as a function of relative exposure thresholds, using the Boeing CFD data. Curves are presented for no intervention, universal masking, vacant middle seating, and both together

Exposure reductions by themselves provide only partial risk information when a virus has an infectivity threshold. The exposure results, when placed in the context of a range of notional infection thresholds, show vacant middle seating, masking, and these when combined reduced the number of infections in a highly variable manner. Figure 4 shows, not surprisingly, the lowest thresholds have all passengers above and the highest thresholds have all passengers below, although the values of these extrema depend on whether VMS or masking were applied. For intermediate thresholds, masking (yellow) had a larger protective effect than VMS (orange), and masking and VMS together (gray) had a larger impact than either alone. The curve for both interventions combined is close to the masking curve, but due to their steepness, a significant number of passengers are impacted by the VMS effect additional to masking, in this hypothetical scenario.

### 3.6 Infection probability

**FIGURE 5:**
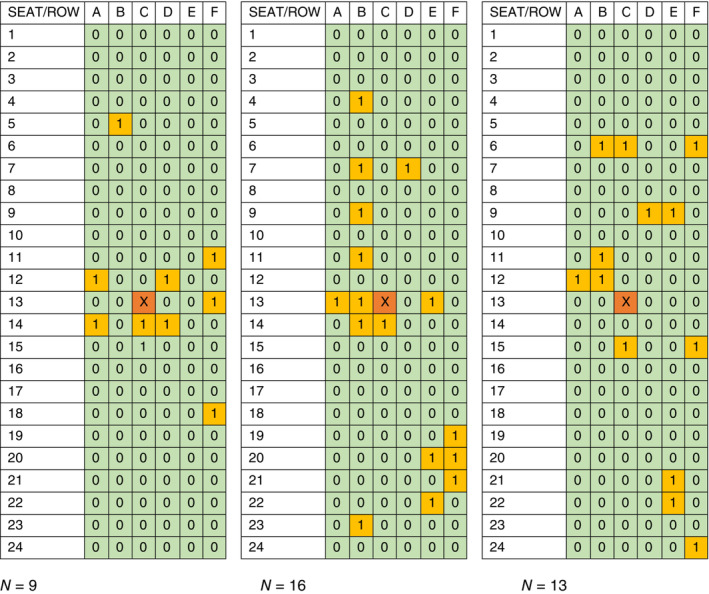
Example patterns of notional infections after applying a random factor to the Wells–Riley infection probabilities, when an *N* of 10 infections were predicted by Equation ((9). Predicted infections are indicated by “1” and highlighted in yellow. The source location is indicated by “X” and highlighted in orange

The number of passengers potentially infected by one or two infectious passengers on board was estimated using the Wells–Riley equation and the average reductions for the three datasets (Table 2). The pattern shown by Figure 4 is largely repeated here. The largest reductions in probable cases belong to masking, the VMS effect is clear but smaller, and the combined effect is only somewhat larger than masking by itself. For higher infectivities, though, this small proportional difference would mean from 1.4 to 7 fewer cases. Figure 5 demonstrates how specific infection patterns that adhere to the Wells–Riley infection probability and the isotropic decrease with distance at the heart of this study can vary widely and appear not especially source‐related, when a random yet proportional factor is applied. The three examples in Figure 5 show 9, 16, and 13 infections, when 10 are predicted as a baseline. Two of these indicate no infections in one of the rows adjacent to the source row. Noteworthy is that the estimates of actual number of infections in the coach section reported independently by Wang et al.,^25^ in their analysis of the TRANSCOM data, are within our notional range of 1 to 25 for no intervention. In their 20 quanta/h scenario for a 12‐h flight and their 100 quanta/h scenario for a 2‐h or a 12‐h flight, their expected numbers are 2.1, 1.9, and 8.1. For a 12‐h flight, nearly all their estimates of infections when masked, from 0.07 to 4.9, are within our masked range of 0.37 to 9.9. In general, the estimates of the current study overlap with Wang in their severe scenario of 100 quanta/h.

**TABLE 2:** Estimated infection reductions for interventions

	Probable infections per infectious passenger					
Number of infectious passengers onboard	One			Two		
No intervention	1.0	10.0	25.0	1.0	9.9	23.2
Masking	0.37	3.76	9.89	0.38	3.82	9.84
VMS	0.62	6.24	15.63	0.64	6.15	14.54
Both	0.24	2.45	6.44	0.24	2.47	6.39
	% Reductions			% Reductions		
No intervention	0.0	0.0	0.0	0.0	0.0	0.0
Masking	63.4	62.4	60.4	63.2	61.2	57.7
VMS	37.7	37.6	37.5	37.7	37.6	37.5
Both	76.3	75.5	74.2	76.2	74.9	72.5
	Infections prevented			Infections prevented		
No intervention	0.0	0.0	0.0	0.0	0.0	0.0
Masking	0.6	6.2	15.1	0.6	6.0	13.4
VMS	0.4	3.8	9.4	0.4	3.7	8.7
Both	0.8	7.6	18.6	0.8	7.4	16.9

## 4 DISCUSSION

The reduction of 38% when a surgical mask was worn by the particle source manikin in the TRANSCOM study is smaller than the 66% reported for surgical mask material in Hao which, in turn, is smaller than the 72% reported in Lindsley et al.^37^ for surgical masks worn by both source and receiving manikins in a conference room setting. Clearly, mask leakage reduces effectiveness, and universal masking is more effective than source‐only application. Liu et al.,^38^ in a paper on mixing and displacement ventilation and masking found that the aircraft cabin average infection risk (as defined in their paper) was 0.20 for no masking, 0.07 for infectious passenger masking, and 0.02 for universal masking, that is, 65% or 90% reductions when the index or all passengers were masked. Note that using Liu's 65% for both *R*_*S*_ and *R*_*R*_ in Equation (8) results in an 88% equivalent reduction for universal masking, that is, approximately 90%. A second Lindsley paper^39^ shows decreasing exposures ordinal to: no masking, receiver masking, source masking, and universal masking, although receiver masking delays the exposure and reduces it early in the emission event compared to source masking. Wang et al.^25^ report 73% or 32% decreases in infection probability (rather than exposure level) when all passengers continuously wear high (65.6%) or low (31.0%) efficiency masks. Case studies of COVID‐19 infection on flights with mandated mask wearing^40^, ^41^ suggests that some virus aerosol is emitted from infectious and inhaled by other masked passengers, and that distance remains an important infection determinant in a masked environment. Results of these studies show that masking alone seems to not eliminate all airborne exposures to infectious droplets and aerosols. This fact points toward multicomponent prevention strategies as good practices.

The three studies analyzed here showed a general decrease in aerosol concentration with distance from a source, although individual measurements showed both high variability close to the emission sources and some countertrend increases in aerosol concentration with distance. This trend broadly agrees with investigations of SARS‐CoV‐2 infection on international flights and high‐speed trains that found seating proximity strongly associated with infection risk: in one instance, 75% of infected passengers were seated within two rows of the symptomatic passenger who likely originated the outbreak^2^ and in another case study, the seats immediately adjacent to the index cases had a higher risk than other seats, with a relative risk (RR) of 27.8 (95% CI 14.4–53.7) on airplanes and 15.7 (95% CI 9.7–25.5) on trains.^42^ However, only one of the three randomly generated infection patterns in Figure 5 adheres to the common understanding that most cabin infections occur within two seat rows of the index pax. Olsen's study of SARS1 observed infections several rows distant from the index.^4^ One study concluded that the risk of COVID‐19 infection on an aircraft is low, even with infectious people onboard.^43^ At least qualitatively, the random infection patterns generated in the present study (Figure 5) appear to be more dispersed than is seen in most of the published infection cases. The infection probabilities and exposure patterns in the present study are based on aerosol measurements made at a distance of at least 0.5 m. Even though the NIOSH bacteriophage experiments had a spray source that included droplets, the lack of measurements at very close range favored measurement after droplets had evaporated into aerosols. Inclusion of large droplets would be expected to result in more infections at shorter distances.

None of the three datasets contain information about the level of absolute exposure risk. In the TRANSCOM and Boeing publications, the cabin particle counts and mass are expressed as a percentage of the total released^24^, ^26^ that are inhaled at given locations. This normalization makes sense for particle tracking and can be used for relative exposure evaluation but must be interpreted correctly in terms of exposure reduction and infection risk. Whenever particles are released into a volume, the number that would be measured at any specific location is much smaller than the number released. Even a person co‐located with the source would inhale a very small fraction of the aerosols expelled. Thus, reductions to a small fraction of the total generated are expected and would apply in most ventilated environments, but these small numbers do not imply small exposure. In broad strokes, aircraft cabin ventilation rates (per passenger) are typically 100 times greater than human respiration rates, meaning the environmental control system reduces exposure by perhaps 99% as a cabin average. Masking can be viewed as reducing the exposure that remains by about 60% and vacant middle seats by on average 37%. The importance of good ventilation in reducing exposure should not be undervalued. Still, a 99% or any other level of exposure reduction is not an indication of the presence or absence of infection risk. The TRANSCOM data were reported down to 0.0000%. However, 0.0001% is still 180 of the approximately 180 million particles released, that is, one millionth. This reporting may be reasonable, however, given experimental uncertainty.

Considering the uncertainties in the infectious fraction of the flying population and in their infectivities, the results are summarized in Table 2 as the probable number of infections per infectious passenger. Perhaps not surprisingly, the relative reduction is nearly independent of the infectivity. That is, VMS reduces the probable infections by about 38%, masking by about 62%, and both combined by about 75%, with only small variations from these numbers due to the infectivity. Table 2 also shows that the infections generated per infectious person change very little with two infectious passengers in the same cabin except at the very high infectivity. For two infectious passengers, then, the number of secondary infections almost doubles—almost, because two infectors might infect the same passenger. The multiplicative effect would decrease further with more infectious passengers. With three infectious passengers, the multiplier is less than three. These fractions are more meaningful when they translate into numbers of people. In the case of vacant middle seats without masking, the lowest % reduction, Table 2 shows nine prevented infections for the highest infectivity of 25 probable infections for one infectious passenger. For the combination of masking and VMS, at the lowest infectivity of one probable infection, these measures are predicted to prevent approximately one infection—essentially the difference between an in‐flight infection occurring and not.

These numbers were generated for very specific example cases, for example, an infected person in an aisle seat near the middle of the cabin. Hundreds of possible cases could be examined and, in the case of two or more infected passengers, thousands of cases. Nevertheless, the consistency of the relative reductions gives some confidence that the results are robust.

The findings in this report have some limitations. The TRANSCOM and Boeing studies did not involve a live virus, meaning that viability in the cabin environment was not considered, and the NIOSH emission source did not represent the variety of respiratory events that could transmit virus (e.g., talking, coughing, sneezing and each of these occurring with varying face covering practices). The NIOSH data were collected in laboratory cabin environments rather than onboard fully operating actual airplanes. Passenger movement during boarding and deboarding and lower ventilation during ground operations were not considered. Even so, from a dose perspective, the in‐flight portion might be the most significant due to much longer exposure duration.^19^ The masking data used in the models did not consider behavioral aspects such as doffing while eating and drinking and noncompliance. Groups having similar immune histories, for example, families were not considered, where spreading their seating to create distance between them would not have a benefit. Finally, the present study did not measure COVID‐19 infection in aircraft cabins, although relative exposure—the %‐difference between exposures in different scenarios—provides useful information. The epidemiological and probabilistic papers cited in the current study support the likelihood that COVID‐19 infection has occurred during commercial passenger flights and, like the analyzes presented here, the dependence of virus exposure on distance. The infection thresholds and infection probabilities examined herein simply cover a reasonable range and are not tied to virus experiments.

## 5 CONCLUSION

Tracer particle data reported by the U.S. Transportation Command (TRANSCOM) and CFD simulations reported by Boeing were used along with NIOSH data, to estimate airborne virus exposure and infection reductions when middle seats are vacant compared to full occupancy and when passengers wear surgical masks in aircraft. Nonlinear regression models that estimate exposure at given distances from the viral source were applied to evaluate exposure reductions from vacant middle seats. Reductions averaged 54% for the seat row where an infectious passenger is located and 36% for a 24‐row cabin containing one infectious passenger. Direct comparison of the source adjacent seat and one set farther away predicted reductions of 20% and 41%. Analysis of the TRANSCOM data showed that universal masking (surgical masks) reduced exposures by 62%.

The extent to which exposure reduction decreases infection risk is not yet fully understood. However, an adaptation of the Wells–Riley equation allowed estimates of probable infections for a range of infectivity. This assessment provided reasonable estimates of relative reduction in the number of infections due to aerosol exposure when masks are worn and middle seats left vacant, although neither the infectious fraction of passengers nor their infectivity are accurately known. For a notional scenario involving 10 infectious passengers compared with no intervention, masking, distancing, and both would prevent 6.2, 3.8, and 7.6 secondary infections, respectively. Across a wide range of scenarios, masking reduced infections by 57%–63%, vacant middle seats by 37%–38%, and both combined by 73%–76%. A vacant middle seat policy comes with the cost of reduced passenger capacity, and masking puts stress on the relationship between airlines and passengers. This assessment shows that these measures, individually and combined provide options to substantially reduce the relative number of infections from aerosol exposure.

## AUTHOR CONTRIBUTIONS

**James S. Bennett:** Conceptualization (equal); formal analysis (equal); methodology (equal); resources (equal); writing – original draft (equal). **Seif Mahmoud:** Data curation (lead); formal analysis (equal); methodology (equal); writing – original draft (equal). **Watts L. Dietrich:** Conceptualization (equal); data curation (supporting); formal analysis (equal); methodology (equal); writing – original draft (equal). **Byron W. Jones:** Formal analysis (equal); methodology (equal); supervision (equal); writing – review and editing (equal). **Mohammad H. Hosni:** Formal analysis (equal); methodology (equal); resources (equal); supervision (equal); writing – review and editing (equal).

## CONFLICT OF INTEREST

The authors declare no potential conflict of interest.

### PEER REVIEW

The peer review history for this article is available at https://publons.com/publon/10.1002/eng2.12582.

## Data Availability

The data that support the findings of this study are openly available at the URLs below [All accessed August 30, 2022]. Readers are encouraged to contact the corresponding author for further information and clarification. “TRANSCOM/AMC Commercial Aircraft Cabin Aerosol Dispersion Tests”^23^ is found at URL: https://www.ustranscom.mil/cmd/docs/TRANSCOM%20Report%20Final.pdf. The TRANSCOM test data spreadsheets are available here: https://figshare.com/search?q=USTRANSCOM. Examples of specific spreadsheets: https://figshare.com/articles/dataset/USTRANSCOM_767‐Flight_Master_Spreadsheet/13093055, https://figshare.com/articles/dataset/USTRANSCOM_777_Hangar_Master_Spreadsheet_with_raw_data/13537379. The journal article version of the TRANSCOM study, “Aerosol tracer testing in Boeing 767 and 777 aircraft to simulate exposure potential of infectious aerosol such as SARS‐CoV‐2”^24^ is found at: https://journals.plos.org/plosone/article?id=10.1371/journal.pone.0246916#sec018. Computational fluid dynamics modeling of cough transport in an aircraft cabin^26^ is found at URL: https://www.nature.com/articles/s41598‐021‐02663‐8. Our analysis of this paper (Ref. 26) used an Excel spreadsheet in their data section, “Supplementary Information 2.” The direct link to their spreadsheet is: https://static‐content.springer.com/esm/art%3A10.1038%2Fs41598‐021‐02663‐8/MediaObjects/41598_2021_2663_MOESM2_ESM.xlsx. The CDC MMWR on MS2 virion dispersion tests and vacant middle seats in aircraft cabins: https://www.cdc.gov/mmwr/volumes/70/wr/mm7016e1.htm.
